# Hepatocellular Carcinoma and Antibody Drug Conjugates: A Systematic Review

**DOI:** 10.7759/cureus.82912

**Published:** 2025-04-24

**Authors:** Srivarshini Maddukuri, Ryan R Haddad, Naga Spandana Battula, Timmie Chay, Tirath Patel, Nabina Dumaru, Lubna Mohammed

**Affiliations:** 1 Internal Medicine, Dr. D.Y. Patil Medical College, Hospital and Research Centre, Pune, IND; 2 Clinical Research, California Institute of Behavioral Neurosciences & Psychology, Fairfield, USA; 3 Medical School, American University of Antigua, St. John, ATG; 4 Department of Radiology, Frimley Park Hospital, Camberley, GBR; 5 Internal Medicine, Dr VRK Women's Medical College, Hyderabad, IND

**Keywords:** antibody-drug conjugate, hepatitis b (hbv), hepatitis c virus (hcv), hepatocellular carcinoma (hcc), liver cancer directed therapies

## Abstract

Hepatocellular carcinoma (HCC) is a leading cause of cancer-related deaths, primarily associated with liver cirrhosis from factors like hepatitis B virus (HBV), hepatitis C virus (HCV), alcohol abuse, metabolic syndrome, and genetic disorders. With the rising incidence of liver cancer, particularly in HBV-endemic regions, research into novel therapies like antibody-drug conjugates (ADCs) has gained momentum. ADCs target cancer cells by attaching cytotoxic drugs to antibodies, minimizing damage to healthy tissue. Recent clinical trials have demonstrated that ADCs targeting GPC3, such as GC33 and 32A9, show promising results in reducing tumor growth and improving patient outcomes in advanced HCC. These therapies offer a potential alternative to conventional chemotherapy, marking a significant advancement in precision oncology. This systematic review was implemented using various databases like PubMed, Google Scholar, Science Direct, EBSCO, and Public Library of Science (PLoS) using regular keywords and MeSH keywords. Eligibility criteria were restricted to free full texts in the English language, humans, and publications between 2019-2024. The exclusion criteria included languages other than English and publications before 2019. A total of 26 articles were identified, and 12 articles were selected after quality assessment.

## Introduction and background

“Is Life worth living? It all depends on the liver.” - William James

Primary liver cancer ranks as the sixth most prevalent cancer worldwide and is the second highest contributor to cancer-related deaths [1]. Approximately 80% of primary liver cancers are HCCs, making the prevalence of HCCs a significant worldwide health concern [2]. The frequency of HCC has been steadily increasing over the past two decades, partly due to the growing rates of obesity and type 2 diabetes, both of which are contributing factors to the development of HCC. Additionally, the onset of HCC is strongly linked with liver cirrhosis, which can result from prolonged and excessive alcohol consumption, exposure to aflatoxin-contaminated foods, and infection with HBV and HCV. The prevalence of these viruses in certain developing nations makes HCC around five times more common there compared to developed regions [2]. In Western countries, the rising number of cases can also be attributed to a delay in diagnosing and treating people who were infected with HCV during the epidemics of the 1970s and 1980s. As a result, these individuals are now developing liver cirrhosis, which increases their risk of liver cancer [3].

The idea of a "magic bullet" that would deliver a medication directly to a predetermined target while avoiding healthy tissue was first conceived by Paul Ehrlich [4]. For many decades, chemotherapy has been the primary treatment option and has been extensively utilized. Nevertheless, the adverse effects and lasting consequences associated with chemotherapy, which result from the nonspecific destruction of cells by cytotoxic agents, have become a major cause for concern regarding its use [4]. Systemic treatments utilizing monoclonal antibodies (mAbs) began to take shape following the introduction of hybridoma technology by Kohler and Milstein in 1975. Greg Winter advanced the field in 1988 by introducing the method for humanizing mAbs, leading to the successful development of therapeutic mAbs for treating different types of cancer [5]. The advancement of more effective anti-cancer therapies depends on merging the targeting ability of antibodies with the strength of small molecules used in chemotherapy, with an inhibitory concentration 50 (IC50) in the sub-nanomolar range. These combined products are classified as a type of anti-cancer medication called antibody-drug conjugates (ADCs) [2].

The treatment of HCC is largely determined by the stage of the tumor. For advanced stages, the only available therapies that provide clinical benefit are systemic treatments, yet the prognosis remains poor [6]. HCC is not simply treated with a single drug, and there is a need to develop new treatment approaches that involve personalized medications [2]. Gemtuzumab ozogamicin, which combines a CD33 antibody with the antitumor antibiotic calicheamicin, was the first licensed ADC in 2000 [7]. It is strongly recommended that ADCs be utilized to target specific antigens found in HCC. In this systematic review, we aim to discuss how ADCs work and their role in HCC, along with current trends in the management of HCC. Although there have been significant advancements in clinical trials and Food and Drug Administration (FDA) endorsements, only a limited number of patients with HCC benefit from immunotherapy. Hence, it is crucial to gain a more comprehensive understanding of the immune regulation mechanisms in HCC and to pinpoint biomarkers that can forecast the response to immunotherapy. This will help direct the clinical application of immunotherapeutic agents and their combinations for the treatment of HCC [8].

## Review

Methods

In accordance with the guidelines outlined in the 2020 PRISMA statement, this systematic review was implemented. Relevant data was collected using advanced search from various databases like PubMed, Google Scholar, Science Direct, EBSCO, and Public Library of Science (PLoS) using regular keywords and MeSH keywords. Eligibility criteria were restricted to free full texts in the English language, humans, and publications between 2019-2024. The exclusion criteria included languages other than English and publications before 2019. 

A PubMed search yielded 551 results, Google Scholar 755 results, ScienceDirect 734 results, EBSCO 672 results, and PLoS 495 results. All the relevant data collected was imported into EndNote. Initially, duplicates were removed, followed by screening articles based on titles and then abstracts. After reviewing the entire article, selected articles were chosen for quality assessment.

Databases, keywords and search strategy, filters, and search results are shown in Table 1.

**Table 1:** Databases, keywords and search strategy, filters and search results used in this systematic review

Databases	Keywords and search strategy	Filters	Search results
PubMed	Hepatocellular Carcinoma OR Liver Cancer OR Liver Tumor OR ( "Liver Neoplasms/complications"[Mesh] OR "Liver Neoplasms/diagnosis"[Mesh] OR "Liver Neoplasms/drug therapy"[Mesh] OR "Liver Neoplasms/etiology"[Mesh] OR "Liver Neoplasms/mortality"[Mesh] OR "Liver Neoplasms/pathology"[Mesh] OR "Liver Neoplasms/surgery"[Mesh] OR "Liver Neoplasms/therapy"[Mesh] ) AND Antibody Conjugate Drugs OR Immunoconjugates OR "Immunoconjugates/administration and dosage"[Mesh] OR "Immunoconjugates/adverse effects"[Mesh] OR "Immunoconjugates/drug effects"[Mesh] OR "Immunoconjugates/metabolism"[Mesh] OR "Immunoconjugates/therapeutic use"[Mesh] OR "Immunoconjugates/toxicity"[Mesh] )	All types of articles between 2019 - 2024, Free full-text in the English language, In humans	551
Google Scholar	"hepatocellular carcinoma" OR "Liver cancer" AND "antibody conjugate drugs" OR "Immunoconjugates"	Publications between 2019 -2024	755
Science Direct	Efficacy of antibody conjugate drugs in Hepatocellular Carcinoma	Publications between 2019 - 2024 Review and Research articles Subject area: Medicine and Dentistry Open access and open archive	734
EBSCO	"hepatocellular carcinoma" OR "Liver cancer" AND "antibody conjugate drugs" OR "Immunoconjugates"	EBSCO Open Research with Full Text in the English language Peer Reviewed between 2019-2024	672
Public Library of Science (PLOS Medicine)	hepatocellular carcinoma OR Liver cancer AND antibody conjugate drugs OR Immunoconjugates	PLOS ONE Subject: Cancer and Neoplasms Research articles between 2019 - 2024	495

Results

Study Selection and Quality Assessment

A total of 3207 articles were collected from five different databases: PubMed, Google Scholar, ScienceDirect, EBSCO, and PLoS. After importing them into Endnote, 11 duplicates were removed. Out of 3196 articles, 26 were retrieved after thorough screening by titles and abstracts and a complete review of the articles. Eight reports were not retrieved, leaving 18 articles for quality assessment.

Quality assessment was performed using AMSTAR 2 (Assessment of Multiple Systematic Reviews), SANRA (Scale for the Assessment of Narrative Review Articles), NOS (The Newcastle- Ottawa Scale), and Cochrane risk bias tool 2.0, leading to the inclusion of 12 finalised articles with scores above 70%.

**Figure 1:**
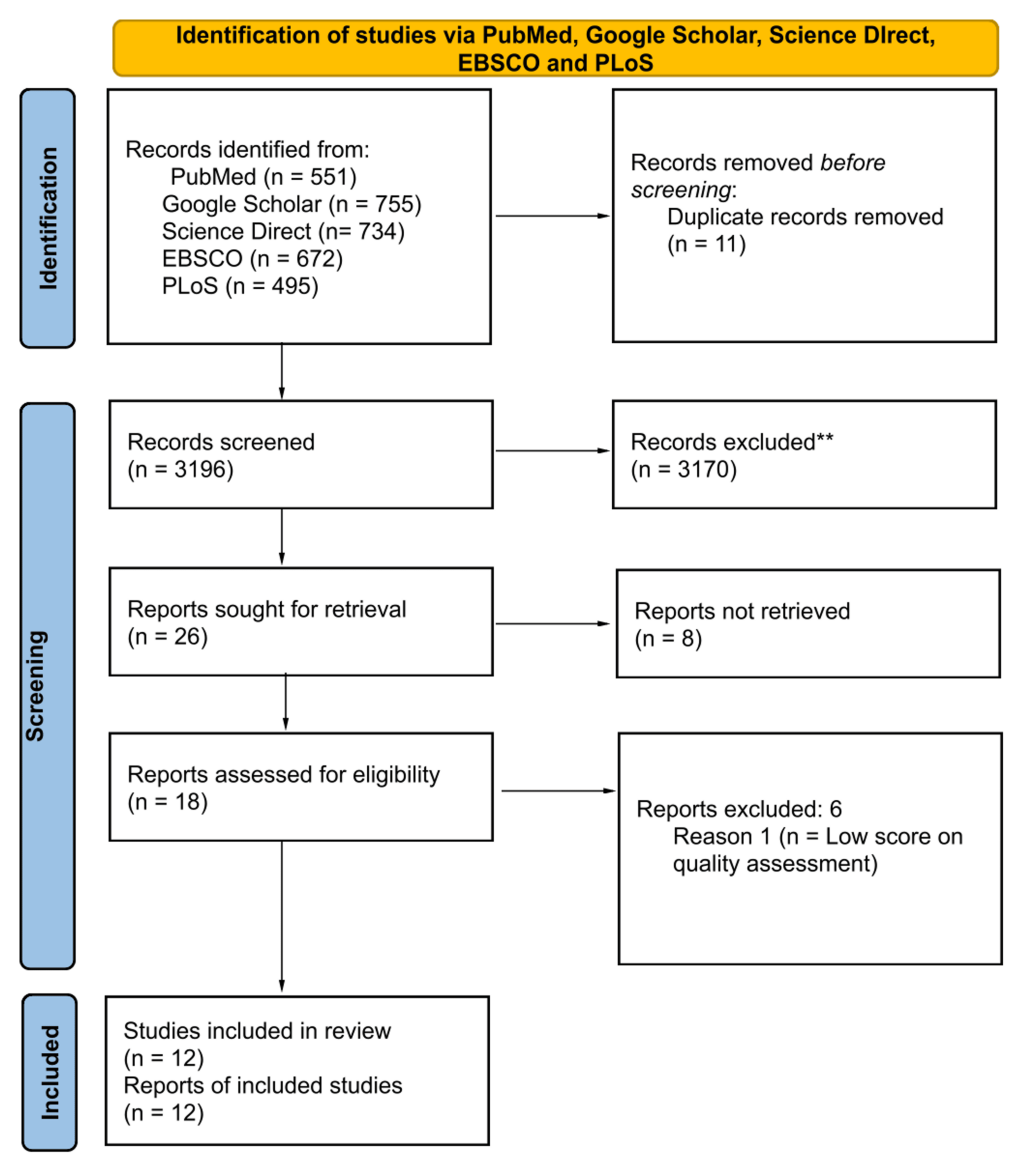
PRISMA flowchart PRISMA: Preferred Reporting Items for Systematic Reviews and Meta-Analyses [9]

The Preferred Reporting Items for Systematic Reviews and Meta-Analyses (PRISMA) flow diagram for search databases is shown in Figure 1, and the tools used for the quality assessment of studies are shown in Table 2.

**Table 2:** Selected studies and their scores for this systematic review using quality assessment tools SANRA: Scale for the Assessment of Narrative Review Articles; AMSTAR: Assessment of Multiple Systematic Reviews

Study no.	Study name	Type of Study	Quality assessment tool used	Score (Acceptable score >70%)
1.	Charles et al. [1]	Review	SANRA	75%
2.	Dahlgren et al. [2]	Review	SANRA	75%
3.	Dasgupta et al. [3]	Systematic review and meta-analysis	AMSTAR 2	81.25%
4.	Filis et al. [4]	Systematic review	AMSTAR 2	78.57%
5.	Hafeez et al. [5]	Review	SANRA	75%
6.	Laface et al. [6]	Review	SANRA	75%
7.	Shastry et al. [7]	Review	SANRA	75%
8.	Shen et al. [8]	Review	SANRA	75%
9.	Tümen et al. [10]	Review	SANRA	83.33%
10.	Valery et al. [11]	Review	SANRA	91.66%
11.	Yao et al. [12]	Review	SANRA	75%
12.	Zarlashat et al. [13]	Review	SANRA	75%

Study Characteristics

The study characteristics of the selected 12 articles are shown in Table 3.

**Table 3:** Study characteristics of the selected articles HCC: hepatocellular carcinoma; ICC: intrahepatic cholangiocarcinoma; ADC: antibody-drug conjugates; HBV: hepatitis B virus; AFP: alpha-fetoprotein

Study no.	First author and year	Study type	Results
1.	Charles et al., 2023 [1]	Review	Cancer immunotherapy uses monoclonal antibodies or checkpoint inhibitors to assist the immune system fight malignancies. Liver cancers such as HCC and ICC escape detection through CTLA-4 and PD-1/PD-L1, but drugs like ipilimumab can block this. High CTLA-4 in ICC is linked to worse outcomes.
2.	Dahlgren et al., 2020 [2]	Review	Optimizing ADC design improves tumor targeting and penetration. GPC3- and CD24-targeting ADCs show promise in HCC, but poor prognosis and drug resistance remain challenges. Combining ADCs with immune checkpoint inhibitors may enhance outcomes.
3.	Dasgupta et al., 2020 [3]	Systematic review and Meta-analysis	Liver cancer rates are rising in Western nations, especially among middle-aged adults, while slowing in some Asian countries. Although HBV declines may reduce HCC deaths over time, global obesity and metabolic risks are emerging as major threats.
4.	Filis et al., 2023 [4]	Review	ADCs are used for solid tumors like breast and lung cancers but may cause toxicity. Research focuses on improving efficacy and reducing resistance. ADCs such as trastuzumab emtansine (T-DM1) and trastuzumab deruxtecan (T-DXd) are notable, especially for breast cancer.
5.	Hafeez et al., 2020 [5]	Review	Molecular imaging has become a key tool in drug development, offering real-time tracking of drug distribution, target expression, and pharmacokinetics. Clinical trials, such as those for DMOT4039A and T-DM1, have demonstrated that molecular imaging can predict the responses to ADC therapy, which helps with patient selection. This approach provides valuable insights into drug stability, target expression, and potential toxicity in healthy organs.
6.	Laface et al., 2022 [6]	Review	Bevacizumab improves survival and response in HCC compared to sorafenib, with tolerable side effects. In individuals with AFP, ramucirumab is a moderately beneficial second-line treatment, but it comes with severe side effects. ADCs like T-DXd and enfortumab vedotin show promise for HCC, with ongoing clinical trials.
7.	Shastry et al., 2023 [7]	Review	Although ADCs are helping treat solid tumors, particularly HER2+ breast cancer, their unique toxicities require careful monitoring. To improve efficacy and safety, future research will focus on overcoming resistance, combination therapies, and next-generation treatments.
8.	Shen et al., 2022 [8]	Review	Atezolizumab and bevacizumab are the top first-line treatments for HCC, but their efficacy is limited by hepatic immunosuppression, requiring better biomarkers. Future studies will concentrate on improving and integrating existing treatments, such as CAR T-cell therapy, oncolytic immunotherapy, and HCC vaccines, which are currently in the early stages of development.
9.	Tumen et al., 2022 [10]	Review	Treatment for advanced stages of HCC, such as resection, ablation, and systemic therapy, is guided by the Barcelona Clinic Liver Cancer (BCLC) classification. Patients in the early stages who meet MILAN criteria are considered for liver transplantation. The necessity for focused therapeutics is highlighted by the lack of reliable indicators for therapy response.
10.	Valery et al., 2022 [11]	Review	The usual first-line treatment for advanced HCC is atezolizumab–bevacizumab; durvalumab–tremelimumab is a more recent addition. Trials exploring immunotherapies and anti-angiogenic drugs are still ongoing, but they haven't surpassed current benchmarks. Early-stage studies that combine immunotherapy and locoregional therapies are promising.
11.	Yao et al., 2021 [12]	Review	More than 15 DCM-based ADCs are being developed, although none have received clinical approval since their discovery in the 1970s. Future cancer treatment appears promising due to developments in drug delivery, target optimization, and bispecific ADCs.
12	Zarlashat et al., 2024 [13]	Review	In advanced HCC, targeted treatments like Tyrosine kinase inhibitors (TKIs) and Immune checkpoint inhibitors (ICIs) have increased survival rates. Combination treatments like atezolizumab–bevacizumab and nivolumab–ipilimumab have improved the treatment. The goal of ongoing research is to improve combination techniques for increased effectiveness and decreased adverse effects.

Discussion

Etiology

Currently, HCC accounts for approximately 75-90% of primary liver cases. Liver cirrhosis, regardless of its cause (such as metabolic syndrome, HBV infection, HCV infection, alcohol abuse, hemochromatosis, α1-antitrypsin deficiency), is the primary risk factor for the development of HCC [10]. Major contributing risk factors include HBV, which accounts for 54% of cases, and HCV, 31% of cases. p53 mutation due to aflatoxin exposure is found in 30 to 60% of HCC patients exposed to aflatoxin [10]. In combination with chronic HBV infection, exposure to aflatoxin can increase the likelihood of developing HCC up to 60 times compared to individuals not exposed to aflatoxin [10].

Even with mild to moderate alcohol consumption, individuals with chronic HCV infection and liver cirrhosis face a notably higher risk of developing HCC, with a five-year cumulative incidence of 23.8%. Alcohol is a significant risk factor for HCC pathogenesis, even in the absence of pre-existing infections [10]. Metabolic diseases can lead to liver steatosis, which can progress to fibrosis or cirrhosis. The occurrence of HCC rises by as much as 1.5% annually when cirrhosis is present [10].

The incidence of liver cancer among patients with non-alcoholic fatty liver disease (NAFLD) who do not have cirrhosis is increasing. Furthermore, the risk of HCC may be further elevated in patients with the most severe form of NAFLD, known as non-alcoholic steatohepatitis (NASH) related cirrhosis [10]. The occurrence of HCC is closely linked to significant environmental factors, particularly virus-related factors. HBV and HCV viruses integrate their genetic material into the host genome or cause double-strand breaks, leading to various genetic changes. Molecular research has pinpointed the most common alterations in HCC, such as mutations in the TERT promoter, TP53, CTNNB1, and epigenetic abnormalities [10]. Epigenetic alterations contributing to HCC include DNA methylation, histone modifications, and non-coding RNA regulation [10]. In HCC, mutations and epigenetic changes lead to dysregulation of key pathways such as Wnt/β-catenin, JAK/STAT, and PI3K/Akt/mTOR, driving tumor growth and survival [10].

Epidemiology

Primary liver cancer is the sixth most common cancer worldwide and the second leading cause of cancer-related deaths, resulting in 830,130 new fatalities in 2020 [1]. The two primary histological types of liver cancer are HCC, representing roughly 80% of all liver cancer instances, and ICC, accounting for around 12-15% of cases [2,3]. The occurrence of cases in Asian nations continues to be one of the highest globally. In 2018, approximately 25% of all new cases and fatalities occurred in Asia, with China representing about half of the worldwide impact [3]. The variation in these rates is largely due to the geographical variances in HBV infection prevalence. Estimates suggest that the prevalence is approximately 18% in China, compared to less than 1% in the United States of America [3].

Types of HCC Based on Tumor Microenvironment

There are two types of liver cancer worldwide based on the tumor microenvironment. Roughly 20-25% of these cancers are known as "hot" tumors, characterized by a high presence of TCD8+ lymphocytes. These tumors also show high levels of PD-1/PD-L1 and CTLA-4, which are important targets for immunotherapies [11]. The second category of tumors is called "immunologically cold" tumors. In these tumors, the immune microenvironment contains more immunosuppressive cells like Foxp3+ regulatory T-cells and fewer effector TCD8+ cells [11]. The oncological applications of ADCs include treating leukemia, lymphoma, myeloma, breast cancer, urothelial tumors, and other types of tumors. It is clear that ADCs have proven to be a significant and distinct presence in the realm of targeted cancer therapy within the field of biotherapeutics [12]. Our current knowledge of genetic pathways and the study of gene expression has offered a methodical approach to comprehend key metabolic, signaling, and cancer-causing pathways in HCC. Consequently, this has facilitated the identification of specific targets for managing HCC [13].

Antibody Drug Conjugates

More efficient anti-cancer treatments have been developed by combining antibodies with the potency of chemotherapeutic small molecules, known as ADCs. All ADC technologies rely on attaching a cytotoxic drug, known as the warhead, to an antibody using a linker molecule. This antibody selectively binds to an antigen that is highly present on cancer cells or in the tumor microenvironment. Once bound, the ADC is taken inside the cell, releasing the potent cytotoxic drug and effectively destroying the tumor cell [2,14]. ADCs are intricate compounds that need careful consideration of different elements. Choosing the right target, a mAb, cytotoxic payload, and the method of linking the antibody to the payload are crucial factors that determine the safety and effectiveness of ADCs [15]. The first ADC drug, Mylotarg (gemtuzumab ozogamicin), was approved by the FDA in 2000 for treating acute myeloid leukemia (AML) in adults, signaling the start of the era of targeted cancer therapy using ADCs [16-18]. With its expanding range of targets and applications, ADC represents a pioneering advancement in targeted cancer therapy. It is poised to emerge as a viable alternative to conventional chemotherapies in the future [16]. To overcome resistance to chemotherapy and/or biologic therapy, ADCs can employ a strategy of identifying new targets. Conventionally, ADC targets have been antigens with high expression levels on cancer cells and minimal or no expression on normal cells [19]. Antigens that show high levels in tumors and low or no levels in healthy tissues are an ideal target for effective drug delivery while minimizing potential side effects [19].

Components of ADC

The composition of ADC includes an antibody, cytotoxic payload, and chemical linker. An effective ADC drug should remain stable in the bloodstream, accurately target the therapy site, and release the cytotoxic payload near the target (such as cancer cells). Each component can impact the overall effectiveness and safety of the ADC. In general, developing ADC requires careful consideration of these key elements, including selecting the target antigen, antibody, cytotoxic payload, linker, and conjugation methods [16,19]. 

ADC medications use the target antigen expressed on tumor cells as a guide to identify them. It also establishes the method (such as endocytosis) by which lethal payloads are delivered into cancer cells [16]. The target antigen should ideally be widely distributed on the cell surface so that the circulating ADC can attach to it [5]. When the relevant antibody binds to the target antigen, it is suitable for internalization. This allows the ADC-antigen combination to enter cancer cells, where it is transported via the proper intracellular pathway and releases its cytotoxic payload [5,16]. The target antigen should express itself exclusively or preferentially on cancer cells and minimally on healthy tissue in order to reduce off-target damage. Finally, to stop the ADC from being sequestered and/or degrading in the bloodstream, antigens that are prevalent in the bloodstream should also be avoided [5].

Choosing the right antibody for ADC is particularly crucial because it can significantly affect the therapeutic index, pharmacokinetic/pharmacodynamic characteristics, and efficacy. An optimal antibody moiety should have a high binding affinity for the target antigen, as well as effective internalization, low immunogenicity, and a long plasma half-life [5,16]. The primary components of antibodies are immunoglobulin G (IgG) molecules, which have a long half-life in the blood circulation system and a strong affinity. The most widely employed antibody subtype is IgG1, which is also easily generated and has moderately strong ADCC and CDC [5].

The warhead that causes cytotoxicity once ADCs have been internalized by cancer cells is known as the cytotoxic payload. IC50 refers to the concentration of a compound required to inhibit 50% of a specific biological or biochemical activity, with lower IC50 values indicating higher potency. Chemicals employed as payloads in ADCs must have high potency (IC50 in the nanomolar and picomolar range), as only around 2% of ADCs may reach the targeted tumor locations after intravenous injection. Currently, the most common cytotoxic payloads for ADCs include immunomodulators, DNA-damaging compounds, and potent tubulin inhibitors [16].

Linker connects the cytotoxic drug to the antibody. It is crucial for the final therapeutic index of ADCs since it is one of the main elements pertaining to the stability of ADCs and payload release profiles [16]. In order for the ADC to reach the cancer cell undamaged, linkers must keep it stable in the bloodstream. However, once internalized, the ADC must be easily split to release the payload. In general, linkers fall into one of two categories: cleavable or non-cleavable [5,16,20]. Compared to cleavable linkers, noncleavable linkers are more stable due to their stable bonds that are resistant to proteolytic degradation. Noncleavable linkers work by causing the ADC complex to internalize, which is followed by the lysosome's breakdown of the mAb component, which releases a cytotoxic chemical that kills tumor cells. They do not damage healthy cells since they do not release cytotoxic chemicals at off-target locations [15,21]. The main class of ADC linkers comprises cleavable linkers. The primary characteristic of cleavable linkers is their susceptibility to cleavage by particular lysosomal enzymes and environmental variations (such as pH and redox potential) in response to intracellular and external conditions [15].

Mechanism of Action of ADC

After intravenous injection, ADCs can bind to target antigens on the cell surface. ADC-antigen complexes can then be internalized through either antigen-dependent endocytosis or antigen-independent pinocytosis, with clathrin-mediated endocytosis being the predominant mode. Payloads can be released into the cytoplasm by linker cleavage in the chemical and enzymatic environment or lysosomal proteolytic antibody degradation for non-cleavable linkers following intracellular trafficking and processing through endosomal and/or lysosomal pathways that depend on organelle acidification. By causing damage to DNA or preventing microtubule assembly, payloads have cytotoxic effects [22].

Role of ADCs in HCC

Glypican-3 (GPC3), an HCC-selective target, is overexpressed in around 75% of HCC cases and is a recognized clinical hallmark of HCC. A phase II clinical trial has assessed GC33, also known as codrituzumab, a humanized monoclonal IgG1 specific for GPC3, in patients with advanced HCC who had previously advanced on sorafenib [23]. Many surface molecules, including GPC3, asialoglycoprotein receptor (ASGP-R), transferrin receptor (TfR), AF20 antigen, somatostatin receptor (SSTR), and lysosome-associated protein transmembrane 4β (LAPTM4B), are highly expressed on the surface of HCC cells in comparison to normal hepatocytes. Among these surface molecules, ASGP-R, TfR, AF20 antigen, SSTR, and LAPTM4B are unsatisfactory for diagnosing HCC but may be useful therapeutic targets. GPC3 is not expressed in diseased liver cells, such as cirrhosis, fatty liver, or hepatitis, and it is rarely expressed in adults when compared to those molecules [23]. GPC3, which is expressed only on the surface of HCC cells, has emerged as a novel star molecule that is highly correlated with the development and occurrence of HCC. In addition to being a useful biomarker for diagnosis, GPC3 is also a key target for HCC immunotherapy [24]. 

ADC can be used to treat HCC because of GPC3's ability to internalize. The ADC may accompany the protein when it is internalized by normal surface GPC3 recycling, but internalization itself is not triggered by an antibody's binding to GPC3 [2]. In GPC3-positive cancer cells, two humanized anti-GPC3 antibody-drug conjugates (hYP7 and hYP9.1b) in the IgG format have been shown to cause complement-dependent cytotoxicity as well as antibody-dependent cell-mediated cytotoxicity (ADCC) [2]. However, the authors came to the conclusion that liver cancer in humans and animals cannot be cured by bare anti-GPC3 antibodies alone [2]. However, the antibodies' high binding affinity for GPC3-positive cancer cells led to further research into using them to create ADCs. They then tested hYP7 because it showed the highest stand-alone cytotoxicity and the highest affinity for GPC3, making it the most promising candidate for clinical development. Duocarmycin SA and pyrrolobenzodiazepine (PBD) dimer, two DNA-damaging chemotherapeutics presently employed in other ADCs being studied in clinical trials, were combined with hYP7 to form the ADC [2]. To increase the likelihood of successful intracellular drug release, the ADCs were made by joining hYP7 to one of the drugs using a dipeptide linker that was prone to lysosomal cleavage. They were chosen for their strength in anti-tumor response when tested in different HCC cell lines. At picomolar doses, both ADCs killed GPC3-positive cancer cell lines (Hep3B, HepG2, and Huh 7); however, the PBD dimer-based ADC was 5-10 times more effective than the duocarmycin SA-based one [2]. Targeting the cell membrane-bound CD24, a mucin-like molecule that is overexpressed in a variety of human carcinomas, including HCC, is another promising ADC tactic. This was examined by attaching two doxorubicin molecules (G7mAb-DOX) to a CD24-targeting antibody. The ADC was able to inhibit tumor growth, reduce systemic toxicity, and extend the survival of HCC-bearing nude mice even though doxorubicin's potency was low (the IC50 is in the low micromolar range) in comparison to the active drugs in the majority of ADCs [2]. 

GC33 is a humanized, recombinant, high-affinity monoclonal antibody that targets the C-terminus of GPC3. According to preclinical evaluations, GC33 promotes ADCC in a way that is dependent on the antigen. In xenograft models, the antibody also decreased tumor development, with the growth reduction roughly corresponding to the level of cell surface antigen [25]. GPC3 and CD16 levels in circulating immune cells could predict the efficacy of the drug when increasing codrituzumab exposure, according to a recent double-blind, phase II trial of GC33 in 185 patients with chemotherapy-unresponsive HCC. This suggests that precision codrituzumab therapy may have potential for treating HCC from this perspective [25]. 

Another monoclonal antibody, 32A9, inhibited the formation of HCC tumors in mice by selectively targeting the central region of GPC3. Subsequently, this study looked into two immunotherapeutic approaches based on 32A9 that included CAR-T cells and an immunotoxin. While the 32A9-CAR-T cells eliminated the tumor cells in vitro and encouraged the regression of HCC xenograft tumors in vivo, it was shown that the antibody-immunotoxin complex was exclusively lethal to GPC3-positive tumor cells [25]. 

HS20, another human anti-GPC3 monoclonal antibody that binds to the molecule's HS moiety, has been demonstrated to suppress tumor growth and block Wnt signaling. Additionally, this antibody did not cause toxicity to mice. Anti-GPC3 treatment approaches have had little clinical effectiveness, despite GPC3 being a well-characterized HCC-associated antigen [25]. One research study investigated the use of GPC3-specific ADCs for the targeted therapy of HCC. hYP7, an antibody that has a strong affinity for GPC3, was combined with strong payloads that damage DNA, such as PBD dimers and duocarmycin SA. In contrast to GPC3-negative HCC cell lines, the ADCs exhibited picomolar action against GPC3-positive ones. In several preclinical studies, they showed potent anticancer activity in vivo. This method presents a viable way to overcome resistance in HCC treatment [26].

**Table 4:** Overview of GPC3-directed therapeutics ADCC: antibody-dependent cell-mediated cytotoxicity; CDC: complement-dependent cytotoxicity

GPC3-Targeted Antibody Therapies	Target Antigen	Payload/ Modality	Mechanism of Action	Trial Phase	Clinical Trial Details	Preclinical Findings
GC 33 (Codrituzumab)	GPC3	Naked antibody	Promotes ADCC	Phase II	Phase II trial in 185 patients with chemotherapy-unresponsive HCC. GPC-3 and CD 16 levels predicted drug efficacy, suggesting potential for precision therapy [25].	Decreases tumor growth in xenograft models, with ADCC dependent on GPC3. Tumor reduction correlates with surface antigen levels [25].
hYP7	GPC3	PBD dimer or duocarmycin SA	Induces CDC and ADCC	Preclinical	-	In preclinical studies, shown to kill GPC3-positive cancer cell lines (Hep3B, HepG2, Huh 7) at picomolar doses. PBD dimer-based ADC is 5-10 times more effective than duocarmycin SA-based ADC [26].
32A9	GPC3	Immunotoxin / CAR-T	Inhibition of tumor formation; works with CAR-T cells and immunotoxins	Preclinical	-	In preclinical studies, 32A9 inhibited tumor formation in mice and was effective in combination with CAR-T cells and immunotoxins, selectively killing GPC3-positive tumor cells [25].
HS20	GPC3	-	Suppresses tumor growth and blocks Wnt signaling	Preclinical	-	In preclinical studies, HS20 suppressed tumor growth and blocked Wnt signaling. No toxicity observed in mice, but limited clinical effectiveness observed in human studies [25].

Limitations

This systematic review talks about promising treatments like GPC3-targeted ADCs; however, it does not discuss their drawbacks, like possible resistance mechanisms that might show up in clinical settings. Limited discussion is provided on the variability of GPC3 expression across different HCC subtypes and its impact on therapy outcomes. The possibility of combination therapy, such as combining immune checkpoint inhibitors or other modalities with GPC3-targeted ADCs, which could improve overall treatment efficacy, has not been sufficiently investigated.

## Conclusions

In conclusion, GPC3 is a promising target for treating hepatocellular carcinoma (HCC) due to its selective overexpression in cancer cells. Monoclonal antibodies like GC33 and ADCs have demonstrated potential by enhancing immune responses and directly delivering cytotoxic drugs to tumor cells. Although clinical trials have shown some success, challenges such as limited efficacy and systemic toxicity remain. Advanced therapies, including CAR-T cells and immunotoxins, show promise in overcoming these obstacles. With further clinical research and the development of precision medicine, GPC3-targeting therapies could become a valuable treatment option for HCC patients.

## References

[1] Charles J, Vrionis A, Mansur A (2023). Potential immunotherapy targets for liver-directed therapies, and the current scope of immunotherapeutics for liver-related malignancies. Cancers (Basel).

[2] Dahlgren D, Lennernäs H (2020). Antibody-drug conjugates and targeted treatment strategies for hepatocellular carcinoma: a drug-delivery perspective. Molecules.

[3] Dasgupta P, Henshaw C, Youlden DR, Clark PJ, Aitken JF, Baade PD (2020). Global trends in incidence rates of primary adult liver cancers: a systematic review and meta-analysis. Front Oncol.

[4] Filis P, Zerdes I, Soumala T, Matikas A, Foukakis T (2023). The ever-expanding landscape of antibody-drug conjugates (ADCs) in solid tumors: A systematic review. Crit Rev Oncol Hematol.

[5] Hafeez U, Parakh S, Gan HK, Scott AM (2020). Antibody-drug conjugates for cancer therapy. Molecules.

[6] Laface C, Fedele P, Maselli FM (2022). Targeted therapy for hepatocellular carcinoma: old and new opportunities. Cancers (Basel).

[7] Shastry M, Gupta A, Chandarlapaty S, Young M, Powles T, Hamilton E (2023). Rise of antibody-drug conjugates: the present and future. Am Soc Clin Oncol Educ Book.

[8] Shen W, Chen Y, Lei P, Sheldon M, Sun Y, Yao F, Ma L (2022). Immunotherapeutic approaches for treating hepatocellular carcinoma. Cancers (Basel).

[9] Page MJ, McKenzie JE, Bossuyt PM (2021). The PRISMA 2020 statement: an updated guideline for reporting systematic reviews. BMJ.

[10] Tümen D, Heumann P, Gülow K, Demirci CN, Cosma LS, Müller M, Kandulski A (2022). Pathogenesis and current treatment strategies of hepatocellular carcinoma. Biomedicines.

[11] Valery M, Cervantes B, Samaha R (2022). Immunotherapy and hepatocellular cancer: where are we now?. Cancers (Basel).

[12] Yao HP, Zhao H, Hudson R, Tong XM, Wang MH (2021). Duocarmycin-based antibody-drug conjugates as an emerging biotherapeutic entity for targeted cancer therapy: Pharmaceutical strategy and clinical progress. Drug Discov Today.

[13] Zarlashat Y, Abbas S, Ghaffar A (2024). Hepatocellular carcinoma: Beyond the border of advanced stage therapy. Cancers (Basel).

[14] Melgarejo-Rubio G, Pérez-Tapia SM, Medina-Rivero E, Velasco-Velázquez MA (2020). Antibody-drug conjugates: the new generation of biotechnological therapies against cancer. Gac Med Mex.

[15] Khongorzul P, Ling CJ, Khan FU, Ihsan AU, Zhang J (2020). Antibody-drug conjugates: A comprehensive review. Mol Cancer Res.

[16] Fu Z, Li S, Han S, Shi C, Zhang Y (2022). Antibody drug conjugate: the "biological missile" for targeted cancer therapy. Signal Transduct Target Ther.

[17] Coats S, Williams M, Kebble B (2019). Antibody-drug conjugates: Future directions in clinical and translational strategies to improve the therapeutic index. Clin Cancer Res.

[18] Makawita S, Meric-Bernstam F (2020). Antibody-drug conjugates: Patient and treatment selection. Am Soc Clin Oncol Educ Book.

[19] Boni V, Sharma MR, Patnaik A (2020). The resurgence of antibody drug conjugates in cancer therapeutics: novel targets and payloads. Am Soc Clin Oncol Educ Book.

[20] Wolska-Washer A, Robak T (2019). Safety and tolerability of antibody-drug conjugates in cancer. Drug Saf.

[21] Nejadmoghaddam MR, Minai-Tehrani A, Ghahremanzadeh R, Mahmoudi M, Dinarvand R, Zarnani AH (2019). Antibody-drug conjugates: Possibilities and challenges. Avicenna J Med Biotechnol.

[22] Li WQ, Guo HF, Li LY, Zhang YF, Cui JW (2021). The promising role of antibody drug conjugate in cancer therapy: Combining targeting ability with cytotoxicity effectively. Cancer Med.

[23] Bell MM, Gutsche NT, King AP, Baidoo KE, Kelada OJ, Choyke PL, Escorcia FE (2020). Glypican-3-targeted alpha particle therapy for hepatocellular carcinoma. Molecules.

[24] Guo M, Zhang H, Zheng J, Liu Y (2020). Glypican-3: A new target for diagnosis and treatment of hepatocellular carcinoma. J Cancer.

[25] Zheng X, Liu X, Lei Y, Wang G, Liu M (2022). Glypican-3: A novel and promising target for the treatment of hepatocellular carcinoma. Front Oncol.

[26] Fu Y, Urban DJ, Nani RR (2019). Glypican-3-specific antibody drug conjugates targeting hepatocellular carcinoma. Hepatology.

